# Data on (+)-usnic acid: A new application to treat toxoplasmosis

**DOI:** 10.1016/j.dib.2016.06.011

**Published:** 2016-06-18

**Authors:** Kaiwei Si, Linlin Wei, Xiaozhuo Yu, Feng Wu, Xiaoqi Li, Chen Li, Yanbin Cheng

**Affiliations:** a Department of Pathogenic Microbiology and Immunology, School of Basic Medical Sciences, Xi’an Jiaotong University Health Science Center, Xi’an 710061, PR China; b Core Research Laboratory, The Second Affiliated Hospital, Xi’an Jiaotong University School of Medicine, Xi’an 710004, PR China; c Center of Teaching Experiment for Postgraduate in Medicine, Xi’an Jiaotong University Health Science Center, Xi’an 710061, PR China

**Keywords:** *Toxoplasma gondii*, Tachyzoite, (+)-Usnic acid, (+)-Usnic acidliposome, Liposome

## Abstract

*Toxoplasma gondii* pathogen is a threat to human health that results in economic burden. Unfortunately, there are very few high-efficiency and low-toxicity drugs for toxoplasmosis in the clinic. (+)-Usnic acid derived from lichen species has been reported to have anti-inflammatory, antibacterial, anti-parasitology, and even anti-cancer activities. In associated with the published article “Effects of (+)-Usnic Acid and (+)-Usnic Acid–Liposome on *Toxoplasma gondii*” [1], this dataset article provided the detailed information of experimental designing, methods, features as well as the raw data of (+)-usnic acid and (+)-usnic acid–liposome on toxoplasma in vivo and vitro. (+)-Usnic acid may be a potential agent for treating toxoplasmosis.

**Specifications Table**

**Table:** 

Subject area	*Biology*
More specific subject area	*Parasitology*
Type of data	*Figures*
How data was acquired	*Microscope (Olympus, Japan), H-600 Transmission Electron Microscope (Hitachi, Japan)*
Data format	*Raw, analyzed*
Experimental factors	*Infectiousness of toxoplasma in vitro and in vivo in the presence of usnic acid.*
Experimental features	*The virulent RH strain of Toxoplasma gondii was cultured by intraperitoneal inoculation of mice and Rat cardiofibroblasts were prepared for primary cardiomyocyte monolayer cultures from 2 to 4-day-old Sprague–Dawley rats in our laboratory*
Data source location	*Xi’an Jiaotong University Health Science Center. Xi’an, P. R. China.*
Data accessibility	*Data is within this article*

**Value of the data**

• This is the first report about development of (+)-usnic acid against toxoplasma.
• The differential experimental models indicated the potential function of (+)usnic acid and its liposome to toxoplasma tachyzoites.
• Provided a new application guidelines for (+)-usnic acid to treat toxoplasmosis.
• Increased the scope of (+) usnic acid usage.

## 1 Data

**Fig. 1:**
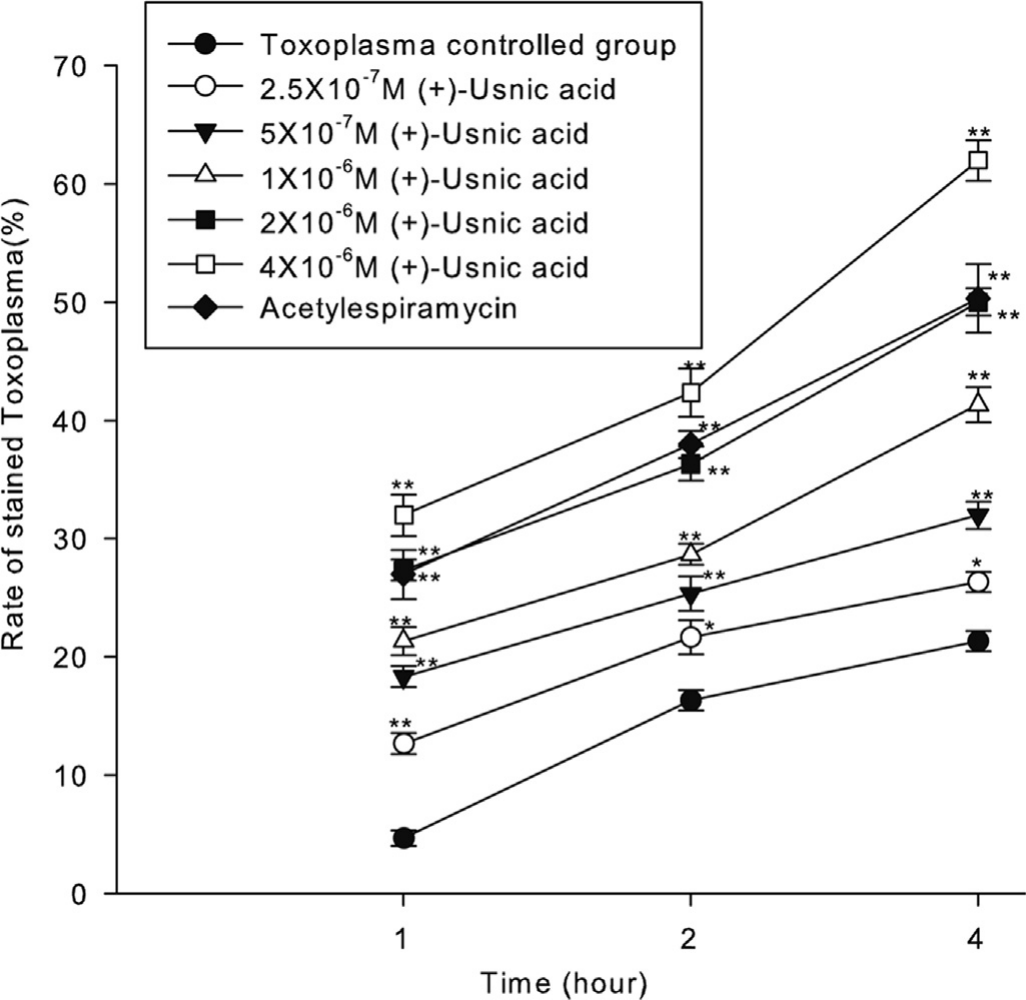
Time- and concentration-dependent relationship of (+)-usnic acid on *Toxoplasma gondii*.

**Fig. 2:**
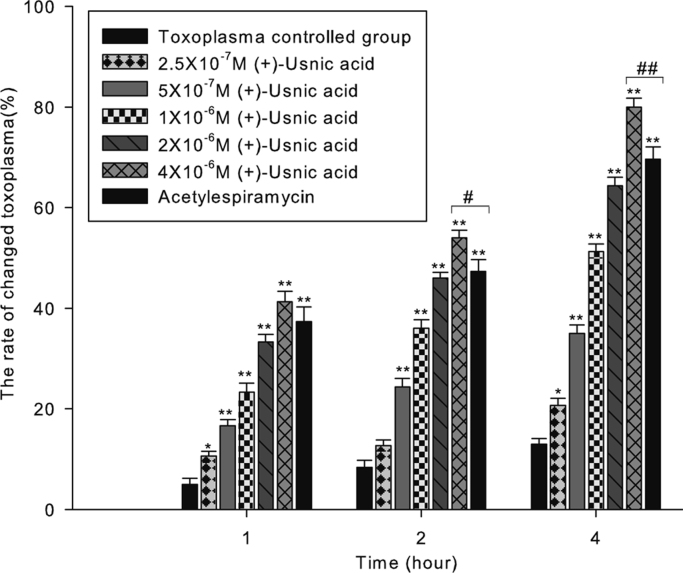
Effects of (+)-usnic acid on *Toxoplasma gondii* tachyzoites with Giemsa staining.

**Fig. 3:**
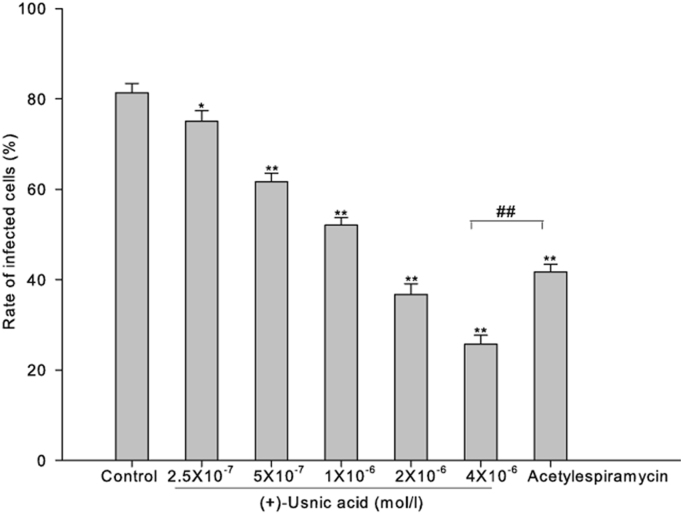
Effect of (+)-usnic acid on the invasion of toxoplasma to cardiofibroblast cells.

**Fig. 4:**
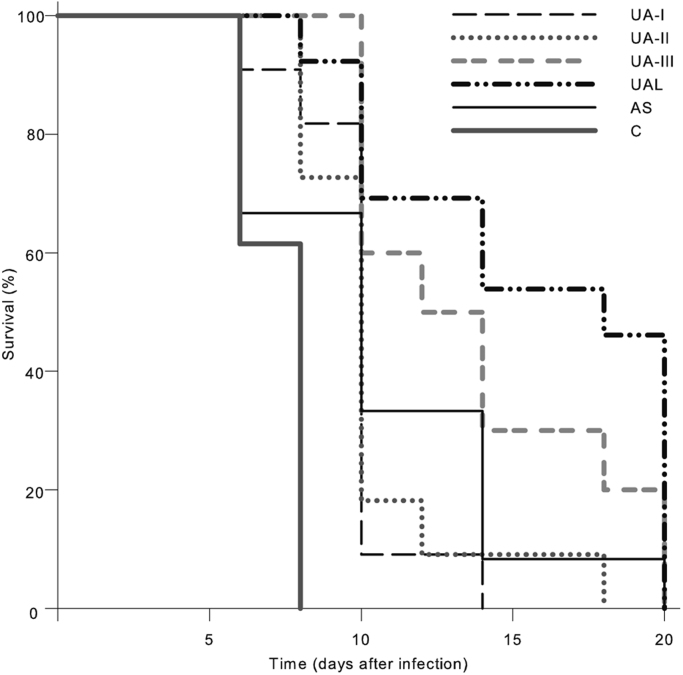
Effect of (+)-usnic acid and (+)-usnic acid–liposome on the acute toxoplasmosis in mice.

**Fig. 5:**
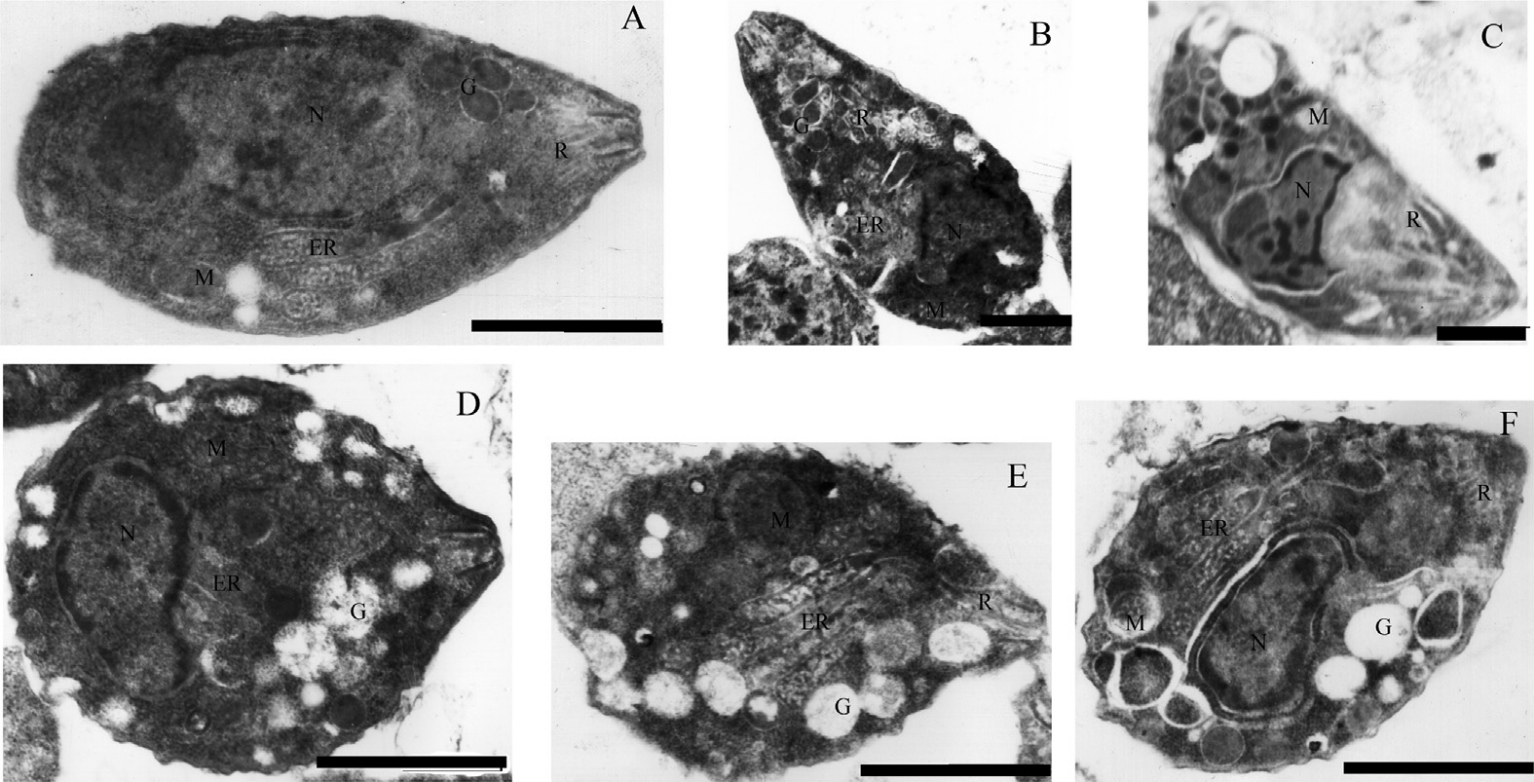
Observation of ultrastructural changes of tachyzoite in mice.

Here, we exemplified the viability of *Toxoplasma gondii* treated by (+)-usnic acid in cell with typan blue and Giemsa staining (Fig. 1, Fig. 2, Fig. 3)and the survival rate of infected mice (Fig. 4) and ultrastructural changes of toxoplasma in vivo (Fig. 5).

## 2 Experimental design, materials and methods

### 2.1 Parasite

The RH strain of *Toxoplasma gondii* was prepared by intraperitoneal inoculation of mice and used in experiments in our laboratory.

### 2.2 Viability and structural changes of toxoplasma treated by (+)-usnic acid

Serial dilutions of (+)-usnic acid (Sigma-Aldrich) were prepared with normal saline in 0.1% Dimethyl Sulfoxide (Sigma-Aldrich). Acetylespiramycin was used as the controlled drug. Toxoplasma tachyzoites (final density 2.56×10^6^/ml) were aliquoted into the treated groups. After treatment for 1 h, 2 h, and 4 h in 25 °C, tachyzoites suspension were respectively smeared and stained with Giemsa in each group. The numbers of changed tachyziotes were counted to calculate the ratio of altered tachyziotes under the light microscope (Olympus, Japan). Meanwhile, tachyzoites suspension were respectively stained with 0.4% trypan blue (Sigma-Aldrich) in each group, and the numbers of colored tachyzoites were counted to calculate the ratio of stained tachyzoites.

### 2.3 Cell culture

Rat cardiofibroblasts were prepared for primary cardiomyocyte monolayer cultures according to a previously published method [2]. Briefly, new born SD rats were sacrificed and the hearts were minced. The tissue was subjected to 3 cycles of proteolytic dissociation by magnetic stirring with 0.125% trypsin (Sigma-Aldrich) solution. The cell pellet was re-suspended in DMEM supplemented. Selective adhesion procedure was performed after the incubation for 1.5 h at 37 °C in a humidified atmosphere (5% CO_2_ and 95% air). The rat cardiofibroblasts were then washed and suspended in medium DMEM.

### 2.4 The invasion of toxoplasma to rat cardiofibroblasts

The cultured rat cardiofibroblasts were infected by tachyzoites simultaneously to investigate the invasion of tachyzoites. Briefly, monolayers of rat cardiofibroblasts were prepared in 24 well culture plates containing cover glasses for 24 h. All cells were divided into seven groups with three wells in each group. The tachyzoites were treated by different final concentrations of (+)-usnic acid and acetylespiramycin for 4 h at 37 °C. Then treated tachyzoites were added to the cells. The cells were continually incubated at 37 °C in a humidified atmosphere (5% CO_2_ and 95% air) for 24 h. Then the cover glasses were taken out, washed with PBS (pH 7.4), and stained by Giemsa dye solution. The numbers of cells infected by tachyzoites were counted to calculate the infection rate.

### 2.5 Preparation of (+)-usnic acid–liposome

(+)-Usnic acid–liposome was prepared with the mechanical dispersion-extrusion method [3]. In briefly, the amount of cholesterol, egg phosphatidylcholine and (+)-usnic acid were dissolved with trichloromethane and dried to prepare liposomal emulsion. The liposomal emulsion was mixed with PBS (pH 7.4) and passed through 200 and 100-nm-pore-size polycarbonate membrane filters for three times. The 120 to 140-nm-size (+)-usnic acid liposome were prepared.

### 2.6 The survival rate of infected mice and the ultrastructural changes of toxoplasma treated by (+)-usnic acid and its liposome

Swiss Webster mice were infected with RH strain tachyzoites of *Toxoplasma gondii* through peritoneal injection. Two hours after inoculation, all the infected mice were administrated orally with drugs. The survival times of mice was observed. In addition, one mouse in each group was killed on the fourth day after infection. The peritoneal exudates of the mouse were harvested. The samples for transmission electron microscope observation were respectively prefixed with 2.5% glutaraldehyde. According to the previously described method [4], ultra-thin-sections (60 nm) were made and observed in an H-600 transmission electron microscope (Hitachi, Japan) for the ultrastructural changes of *Toxoplasma gondii*.

### 2.7 Statistical analyses

Data were expressed as the mean±S.E.M. The statistical significance of the differences between groups was determined by One-Way Analysis of Variance (ANOVA). Survivals of mice were analyzed with Kaplan–Meier with Sigma plot v11.0. Values of *p*<0.05 was statistically considered significant.
