# Supplementary material for: Data on (+)-usnic acid: A new application to treat toxoplasmosis
Source: Data Brief. 2016 Jun 18;8:648–53. doi: 10.1016/j.dib.2016.06.011 (PMC4939410; doi:10.1016/j.dib.2016.06.011)
Supplement: Supplementary file 1 — Supplementary material [file mmc1.doc]

**Conflicts of interest**

Dear editor,

I submit the manuscript titled "Data on (+)-Usnic acid and its liposome against Toxoplasma Gondii in mice and decreasing the invasion of tachyzoite to cardiofibroblasts" to Data in Brief on behalf of all authors, who have read the manuscript and approved the submission and publication.

Here, I declare that this manuscript represents original material that has not been published previously and submitted to any journal else. I also declare that all experimental procedures carried out in this study were performed in accordance with the international, national and institutional rules.

Thank you for considering the manuscript published in Data in Brief in advance.

Sincerely Yours,

Yanbin Cheng

Corresponding author: Prof. Yanbin Cheng,

Post box 64, Department of Pathogenic Microbiology and Immunology, School of Basic Medical Sciences, Xi'an Jiaotong University Health Science Center., 76 West Yanta Road, Xi'an 710061, China. Tel: +86 29 82655187; Fax: +86 29 82655187.

E-mail: chengyb@mail.xjtu.edu.cn
